# Proteome-wide mendelian randomization study implicates therapeutic targets in common cancers

**DOI:** 10.1186/s12967-023-04525-5

**Published:** 2023-09-21

**Authors:** Feihong Ren, Qiubai Jin, Tongtong Liu, Xuelei Ren, Yongli Zhan

**Affiliations:** 1 Guang’anmen Hospital, China Academy of Chinese Medical Sciences, Beijing, 100053 China; 2 Graduate School, Beijing University of Chinese Medicine, Beijing, 100029 China

**Keywords:** Drug target prediction, Cancers, Mendelian randomization, Protein quantitative trait loci

## Abstract

**Background:**

The interest in targeted cancer therapies has been growing rapidly. While numerous cancer biomarkers and targeted treatment strategies have been developed and employed, there are still significant limitations and challenges in the early diagnosis and targeted treatment of cancers. Accordingly, there is an urgent need to identify novel targets and develop new targeted drugs.

**Methods:**

The study was conducted using combined cis-Mendelian randomization (cis-MR) and colocalization analysis. We analyzed data from 732 plasma proteins to identify potential drug targets associated with eight site-specific cancers. These findings were further validated using the UK Biobank dataset. Then, a protein–protein interaction network was also constructed to examine the interplay between the identified proteins and the targets of existing cancer medications.

**Results:**

This MR analysis revealed associations between five plasma proteins and prostate cancer, five with breast cancer, and three with lung cancer. Subsequently, these proteins were classified into four distinct target groups, with a focus on tier 1 and 2 targets due to their higher potential to become drug targets. Our study indicatied that genetically predicted KDELC2 (OR: 0.89, 95% CI 0.86–0.93) and TNFRSF10B (OR: 0.74, 95% CI 0.65–0.83) are inversely associated with prostate cancer. Furthermore, we observed an inverse association between CPNE1 (OR: 0.96, 95% CI 0.94–0.98) and breast cancer, while PDIA3 (OR: 1.19, 95% CI 1.10–1.30) were found to be associated with the risk of breast cancer. In addition, we also propose that SPINT2 (OR: 1.05, 95% CI 1.03–1.06), GSTP1 (OR: 0.82, 95% CI 0.74–0.90), and CTSS (OR: 0.91, 95% CI 0.88–0.95) may serve as potential therapeutic targets in prostate cancer. Similarly, GDI2 (OR: 0.85, 95% CI 0.80–0.91), ISLR2 (OR: 0.87, 95% CI 0.82–0.93), and CTSF (OR: 1.14, 95% CI 1.08–1.21) could potentially be targets for breast cancer. Additionally, we identified SFTPB (OR: 0.93, 95% CI 0.91–0.95), ICAM5 (OR: 0.95, 95% CI 0.93–0.97), and FLRT3 (OR: 1.10, 95% CI 1.05–1.15) as potential targets for lung cancer. Notably, TNFRSF10B, GSTP1, and PDIA3 were found to interact with the target proteins of current medications used in prostate or breast cancer treatment.

**Conclusions:**

This comprehensive analysis has highlighted thirteen plasma proteins with potential roles in three site-specific cancers. Continued research in this area may reveal their therapeutic potential, particularly KDELC2, TNFRSF10B, CPNE1, and PDIA3, paving the way for more effective cancer treatments.

**Supplementary Information:**

The online version contains supplementary material available at 10.1186/s12967-023-04525-5.

## Introduction

Cancer remains a significant global health issue, responsible for millions of deaths annually [1]. Despite significant advancements in medical technology and cancer research [2], the complex molecular characteristics and disease mechanisms of cancer give rise to numerous limitations and challenges in its diagnosis and treatment. Firstly, conventional screening methods, such as imaging and pathology tests, have limited effectiveness in detecting cancer at an early stage [3, 4]. Secondly, for cancers that cannot be fully cured through surgery, traditional drug treatment methods like chemotherapy, immunotherapy, hormone therapy, interferons, and interleukins show unsatisfactory efficacy, along with toxic side effects on normal cells. In recent years, there have been notable advancements in targeted cancer therapies. These therapies employ drugs that specifically target and inhibit molecules or signaling pathways associated with cancer cells, such as tyrosine kinase inhibitors and monoclonal antibodies [5]. Despite the progress made, the diverse subtypes of cancer and the development of drug resistance pose challenges in achieving comprehensive cancer inhibition or cure using a single drug. Consequently, there is an urgent need to develop new strategies for cancer diagnosis and treatment to overcome the limitations encountered in early cancer detection and treatment.

Plasma proteins, as vital constituents of the blood, actively participate in various biological processes within the human body, encompassing signaling, transportation, growth, repair, and infection defense [6]. Notably, plasma proteins are also recognized for their significant role in cancer development and treatment. On the one hand, plasma proteins serve as valuable biomarkers in cancer, enabling early diagnosis, prognosis evaluation, and treatment monitoring [7]. On the other hand, these proteins actively engage in cancer cell growth, migration, invasion, and the creation of the tumor microenvironment. Consequently, they exhibit potential as drug targets [8]. By precisely targeting these proteins, it becomes feasible to effectively counteract tumor cell proliferation and impede tumor progression.

The identification of plasma protein biomarkers and their corresponding targeted drugs has opened up new avenues for precise cancer treatment. However, currently, used plasma protein biomarkers and the drugs developed against them still have limitations. Firstly, some protein markers are expressed in multiple diseases, lacking specificity [9]. Secondly, drug resistance can arise from tumor cell escape mechanisms or target mutations. Thirdly, the development of cancer involves intricate signaling networks, and there are still unidentified protein markers and therapeutic targets [10]. Consequently, there is an urgent need to discover additional plasma protein biomarkers that exhibit greater specificity and sensitivity in assessing cancer risk. By elucidating the correlations of these biomarkers with specific molecular mechanisms and signaling pathways, they could provide crucial insights into targeted cancer treatment. Several preclinical and prospective observational studies have indicated the promise of novel plasma proteins as cancer biomarkers [11, 12]. However, conventional observational designs are susceptible to various biases, such as residual confounding due to unmeasured or imprecisely measured confounders, as well as reverse causation. As a result, significant challenges exist in translating the findings of observational studies into effective strategies for cancer control.

Recently, Mendelian randomization (MR) analysis has become an increasingly valuable approach for drug target development and drug repurposing [13]. This method relies on three core assumptions: (I) the genetic instrumental variables (IVs) are strongly associated with the exposure being investigated (relevance assumption); (II) the IVs are not influenced by confounding factors that affect both the exposure and outcome (independence assumption); and (III) the IVs do not have additional effects on the outcome through pathways other than the exposure being studied (exclusion restriction assumption). Using genetic variants associated with protein levels, referred to as protein quantitative trait loci (pQTL), as instrumental variables in MR analysis offers several advantages with respect to these assumptions [14]. pQTL variants are often derived from population-based genetic studies, such as genome-wide association studies (GWAS), which combine information on both genetic variants and circulating protein levels. By selecting genetic instruments that map closely to the gene of interest (cis-acting variants) rather than those located farther away (referred to as trans-acting variants), violations of the exclusion restriction assumption can be minimized. While MR analysis has successfully identified potential drug targets for various diseases, there have been only a limited number of MR studies that integrate GWAS and pQTL data, specifically in the context of cancer.

**Fig. 1:**
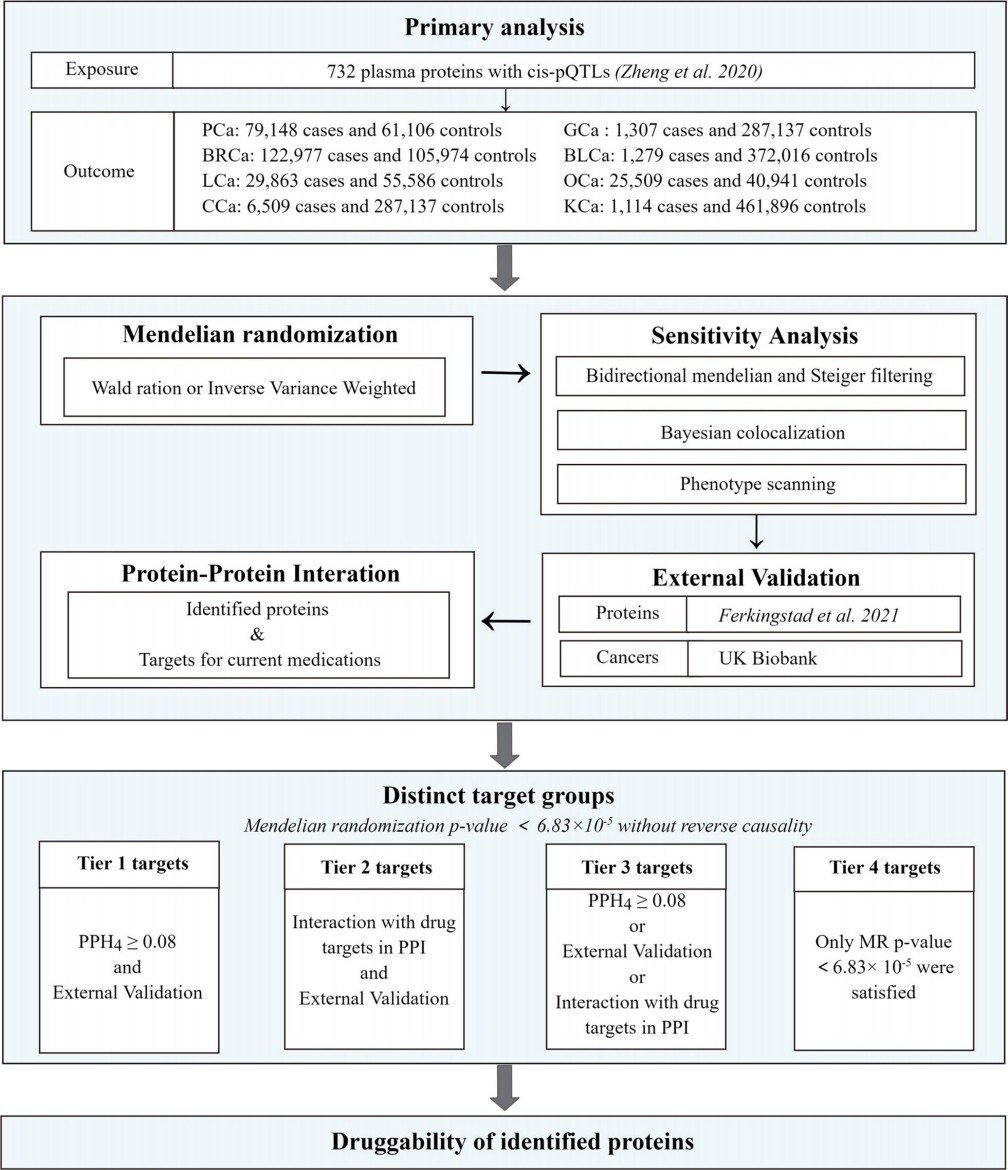
Workflow of Mendelian randomization study revealing causality from plasma protein on site-specific cancers. PPI: protein–protein interaction; PCa: prostate cancer; BRCa: breast cancer; LCa: lung cancer; CCa: colorectal cancer; BLCa: bladder cancer; OCa: ovarian cancer; KCa: kidney cancer; GCa: gastric cancer; cis-pQTL: cis-protein quantitative trait loci; PPH4: posterior probability of hypothesis 4

This study utilized MR analysis to identify potential drug targets among plasma proteins for the eight most common site-specific cancers globally [15]: prostate cancer (PCa), breast cancer (BRCa), lung cancer (LCa), colorectal cancer (CCa), bladder cancer (BLCa), ovarian cancer (OCa), kidney cancer (KCa), and gastric cancer (GCa). We utilized GWAS data for these eight cancers and plasma pQTL data from the study conducted by Zheng et al. [16] to identify plasma proteins that may have causal effects on developing these site-specific cancers. We then employed bidirectional MR analysis, Bayesian colocalization analysis, and phenotype scanning to further validate our findings. In addition, we constructed an interaction network to visually depict the connections among the identified plasma proteins and the targets of current drugs used in the treatment of these site-specific cancers. To ensure the reliability of our conclusions, we externally validated our findings using GWAS data for site-specific cancers from the UK Biobank and the latest published plasma pQTL data [17]. The detailed research workflow is presented in Fig. 1.

## Materials and methods

### Data sources of plasma protein quantitative trait loci

In the primary MR analysis, we obtained plasma pQTL data from the study conducted by Zheng et al. [16]. Zheng's study integrated data from five GWAS sources [6, 8, 18–20]. We selected pQTLs for inclusion in our study based on the following criteria [16]: (I) pQTLs reached the threshold of genome-wide significance (P < 5 × 10^–8^); (II) pQTLs were located outside the major histocompatibility complex (MHC) region (chr6, 26–34 Mb); (III) there was no significant linkage disequilibrium (LD) among the pQTLs (linkage disequilibrium clumping r^2^ < 0.001); (IV) the variants were cis-acting; and (V) the F-statistic for each protein’s pQTL was greater than 10 to minimize bias caused by weak instrumental variables. Ultimately, we included a total of 736 cis-acting single nucleotide polymorphisms (SNPs) representing 732 proteins (Additional file 1: Table S1). For external validation, we obtained protein pQTLs data from the study conducted by Ferkingstad et al. [17]. This study included data from 35,559 participants and evaluated 4907 plasma proteins.

### Data sources of site-specific cancers

In the primary MR analysis, we obtained GWAS data for PCa from the Prostate Cancer Association Group to Investigate Cancer Associated Alterations in the Genome (PRACTICAL). This dataset included 79,148 PCa cases and 61,106 controls [21]. For BRCa, we obtained the largest available GWAS summary data from a meta-analysis of 122,977 BC cases (69,501 ER + BC and 21,468 ER − BC) and 105,974 controls with European ancestry, combining data from the Breast Cancer Association Consortium (BCAC) [22]. The largest available GWAS summary statistics for LCa were derived from the Transdisciplinary Research in Cancer of the Lung and The International Lung Cancer Consortium (TRICL-ILCCO), which included a total of 29,863 cases and 55,586 controls [23]. For CCa, we obtained data from the FinnGen consortium, which included 6,509 CCA cases and 287,137 controls. Similarly, for GCa, we obtained data from the same consortium, consisting of 1,307 cases and 287,137 controls. These data were obtained from publicly available summary statistics (https://r9.finngen.fi/). The GWAS summary statistics for BLCa (1279 cases and 372,016 controls) and KCa (1,114 cases and 461,896 controls) were acquired from The IEU OpenGWAS project (https://gwas.mrcieu.ac.uk/). Regarding overall OCa, we obtained the GWAS summary data from the Ovarian Cancer Association Consortium (OCAC), which included 25,509 ovarian cancer cases and 40,941 controls [24]. Additionally, for external validation of the significant proteins identified in the primary analysis, we obtained summary statistics from the UK Biobank for PCa, BRCa and LCa. Additional file 1: Table S2 lists the sources and corresponding information of all aggregated statistical datasets used in this study.

## Statistical analysis

### Mendelian randomization analysis

In our study, we treated plasma proteins as exposures and the eight site-specific cancers as outcomes. To investigate the causal relationships between the exposures and outcomes, we utilized the “TwoSampleMR” package (Version 4.2.2) in the R program (https://github.com/MRCIEU/TwoSampleMR). We employed the Wald ratio method to generate effect estimates when considering a plasma protein instrumented by a single SNP [25]. We primarily used the Inverse Variance Weighted (IVW) method for proteins instrumented by two or more SNPs [26, 27], followed by heterogeneity analysis. The results were presented as odds ratios per standard deviation increase in genetically determined plasma proteins.

In the primary analysis, we addressed the issue of multiple comparisons by applying the Bonferroni correction. We set a threshold P-value of 0.05 divided by the number of proteins (0.05/732), resulting in a significance threshold of P < 6.83 × 10^−5^. We selected the most significant findings based on this threshold for further investigation. The initially identified proteins were then externally validated using MR, with a P-value threshold of 0.05. To verify the preliminary findings, we employed a homozygous variation strategy. This strategy utilized the same SNPs as the genetic instruments that were used in the primary analysis. Additionally, we employed a significant variation strategy, which utilized genome-wide significant SNPs as genetic instruments [28].

### Steiger filtering and bidirectional Mendelian randomization analysis

In our primary analysis, we implemented Steiger filtering on the proteins identified within three distinct site-specific cancers [29]: prostate, breast, and lung (Table 1). To bolster the dependability of our MR analysis, we adopted genetic instruments pertinent to these three site-specific cancers from the UK Biobank, conforming to the pQTLs selection criteria. These instruments were then deployed in a bidirectional MR analysis to explore potential instances of reverse causality. The threshold for statistical significance was established at a P-value of 0.05. Any plasma proteins from our results that displayed indications of reverse causality were deliberately omitted (Additional file 1: Table S3).

**Table 1:** MR analysis results and reverse causality detection for 16 plasma proteins significant related with cancers

Cancers	Proteins	SNP	Unipoint	OR (95%)	*P*	PVE (%)	Steiger filtering	Bidirectional MR *P*-value (IVW/MR-egger)	F
Prostate cancer	KDELC2	rs74911261	Q7Z4H8	0.89 (0.86, 0.93)	1.89E−08	8.61	8.23E−60	0.189^b^	310.89
	SPINT2	rs71354995	A0A140VJV6	1.05 (1.03, 1.06)	1.49E−06	36.59	NA	0.231^b^	1905.11
	TNFRSF10B	rs4871844	O14763	0.74 (0.65, 0.83)	2.41E−07	0.94	3.19E−06	0.228^b^	30.25
	GSTP1	rs1695	P09211	0.82 (0.74, 0.90)	1.82E−05	1.47	3.14E−10	0.337^b^	49.17
	IGF2R	rs629849	P11717	0.92 (0.90, 0.94)	4.57E−10	18.27	7.55E−138	0.026^b^	738
	CTSS	rs41271951	P25774	0.91 (0.88, 0.95)	2.56E−07	11.33	2.24E−81	0.806^b^	421.77
	HDGF	rs4399146	P51858	1.14 (1.07, 1.21)	5.73E−05	3.01	3.62E−20	0.015^b^	99.26
Breast cancer	CPNE1	rs12481228	Q99829	0.96 (0.94, 0.98)	5.15E−05	16.76	1.24E−130	0.979^b^	664.52
	PDIA3	rs3110081	P30101	1.19 (1.10, 1.30)	3.20E−05	1.14	3.39E−08	0.315^b^	36.75
	GDI2	rs2890364	P50395	0.85 (0.80, 0.91)	3.34E−06	1.75	4.19E−12	0.598^b^	57.03
	ISLR2	rs2959011	Q6UXK2	0.87 (0.82, 0.93)	4.27E−05	1.71	2.19E−12	0.336^b^	57.55
	CTSF	rs1791679	Q9UBX1	1.14 (1.08, 1.21)	7.53E−06	2.26	5.40E−16	0.245^b^	76.25
Lung cancer	SFTPB	rs1130866	P07988	0.93 (0.91, 0.95)	6.36E−09	46.61	3.77E−178	0.771^b^	972.31
	CTSH	rs34593439	P09668	1.07 (1.05, 1.10)	1.71E−08	49.95	1.07E−199	0.002^a^	1098.94
	ICAM5	rs281439	Q8N6I2	0.95 (0.93, 0.97)	2.94E−05	53.15	9.08E−219	0.217^b^	1194.8
	FLRT3	rs11908097	Q9NZU0	1.10 (1.05, 1.15)	2.16E−05	14.37	2.90E−50	0.217^b^	255.54

PVE: proportion of variance explained; IVW: inverse variance weighted; OR: Odds ratios; SNP: single nucleotide polymorphism; a: The P-value of MR-egger; b: The P-value of MR-IVW

### Bayesian colocalization analysis

The intent of colocalization analysis is to determine whether a particular genetic variant is simultaneously associated with both the exposure factor and the outcome through the modulation of gene expression at common loci. This technique is notably advantageous for evaluating exposures like proteins and gene expression, especially when Mendelian randomization analysis focuses on a specific gene region [20]. In our research, we employed the 'coloc' package (https://github.com/chr1swallace/coloc), leveraging Bayesian methods to estimate the posterior probability of a shared causal variant between two traits. The package’s default arguments were adhered to throughout our analyses, which included prior probabilities for variant-trait associations. Assuming a solitary causal variant, four hypotheses can be outlined: H0, proposing the lack of causal variants for both traits; H1, positing the existence of a causal variant for trait 1; H2, suggesting a causal variant for trait 2; H3, postulating two distinct causal variants for traits 1 and 2; and H4, proposing a shared causal variant between the two traits [30]. We considered significant colocalization between two signals to be present when there was strong evidence, denoted by a posterior probability of hypothesis 4 (PPH4) for shared causal variants being ≥ 0.8 [31].

### Phenotype scanning

Within the context of our study, we executed phenotype scanning to investigate the associations of the identified pQTLs with diverse traits. This scanning was carried out using the “phenoscanner” tool [32]. Identified pQTLs that met the following criteria were deemed to possess pleiotropic effects, thus requiring careful interpretation of their implications: (1) an observed association reached genome-wide significance, denoted by P < 5 × 10^−8^; and (2) the pQTLs demonstrated associations with known risk factors pertinent to the respective cancer, such as proteins, genes, or diseases.

### Protein–protein Interaction Network

To delve deeper into the interactions among the identified proteins and to enhance our understanding of the biological processes involving protein regulation, signal transduction, and functional modulation, we constructed a protein–protein interaction (PPI) network in our research. Moreover, in our quest to elucidate the interactions between the identified proteins and the targets of current anticancer drugs, we sourced target information for existing cancer therapeutics from the DrugBank database (https://www.drugbank.ca) [33]. We further gathered information concerning drugs that target the identified proteins. Leveraging this data, we employed the Search Tool for the Retrieval of Interacting Genes (STRING) database, version 11.5 (https://string-db.org/) [34, 35] to construct the protein–protein interaction network. The threshold for the minimum required interaction score was designated as 0.4 [36].

### Classification hierarchy of proteins as potential drug targets

Upon applying the Bonferroni correction and identifying proteins that surpassed the threshold P-value, we stratified these proteins into four distinct target categories. Specifically, tier 1 targets encompassed proteins with robust supporting evidence (PPH4 > 0.8) and successful replication in external validation. Tier 2 targets incorporated proteins that exhibited associations with known drug targets within the PPI network and concurrently met the criteria for external validation. Tier 3 targets consisted of proteins that either boasted a PPH4 > 0.8, met the criteria for external validation, or were associated solely with known drug targets within the PPI network. The proteins not falling into the first three tiers were classified as tier 4 targets.

## Results

In conclusion, our study employed MR analysis to scrutinize the causal relationships between 732 plasma proteins and eight site-specific cancers. At the Bonferroni significance level (P < 6.83 × 10^−5^), the MR analysis unveiled associations between seven proteins (KDELC2, SPINT2, TNFRSF10B, GSTP1, IGF2R, CTSS, HDGF) and PCa; five proteins (CPNE1, PDIA3, GDI2, ISLR2, CTSF) and BRCa; and four proteins (SFTPB, CTSH, ICAM5, FLRT3) and LCa. However, we detected no associations between plasma proteins and CCa, BLCa, OCa, KCa, or GCa. Although Steiger filtering provided assurance on the directionality of the causal relationships, bidirectional Mendelian randomization unveiled reverse causality between IGF2R, HDGF and PCa, as well as between CTSH and LCa. Consequently, we excluded these three proteins from subsequent analyses.

### MR results for site-specific cancers

**Fig. 2:**
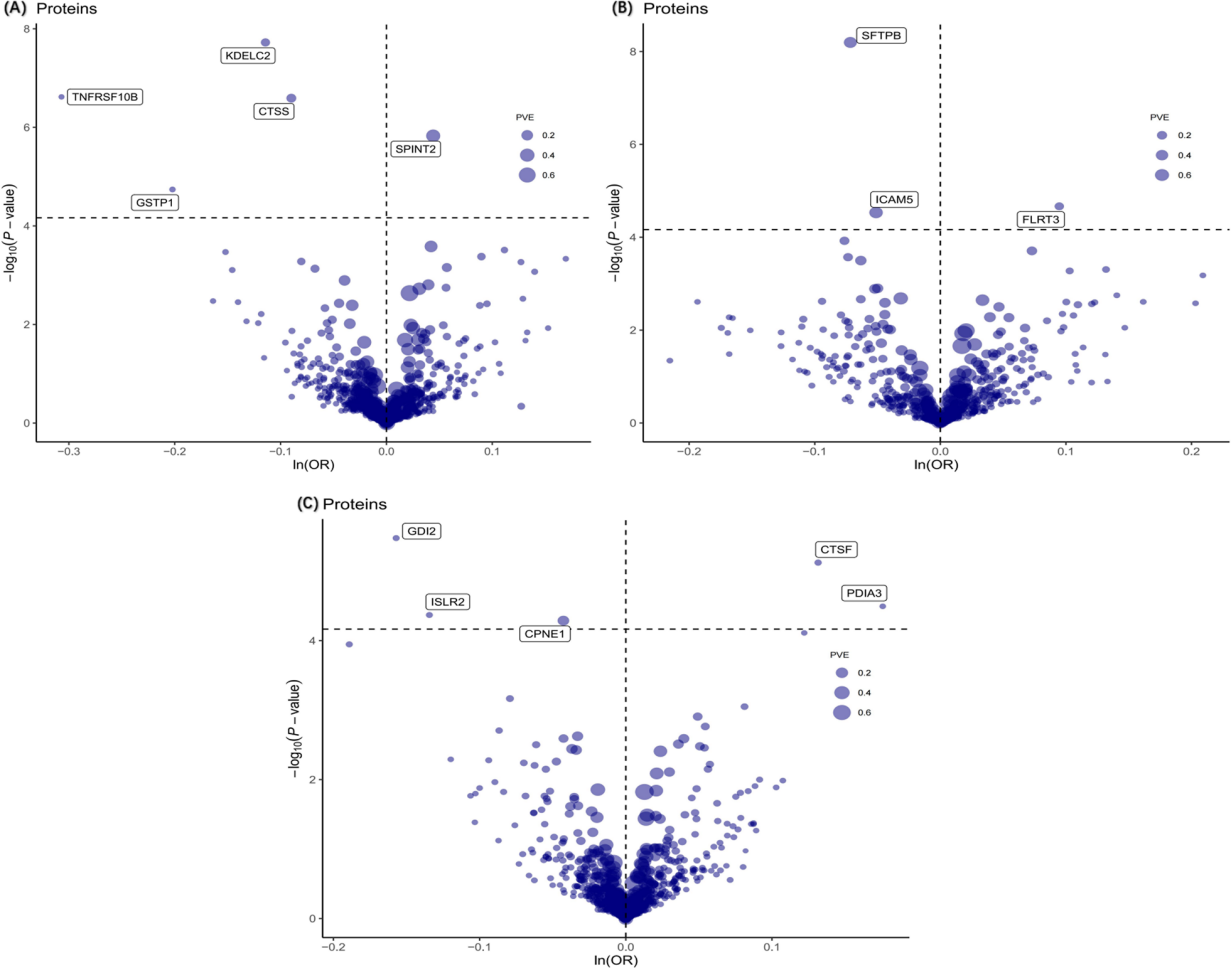
Volcano plots of the MR results. The association between 732 plasma and the risk of **A** prostate cancer, **B** lung cancer, and **C** breast cancer. OR for increased risk of cancers were expressed as per SD increase in plasma protein levels. ln: natural logarithm; PVE: proportion of variance explained

**Fig. 3:**
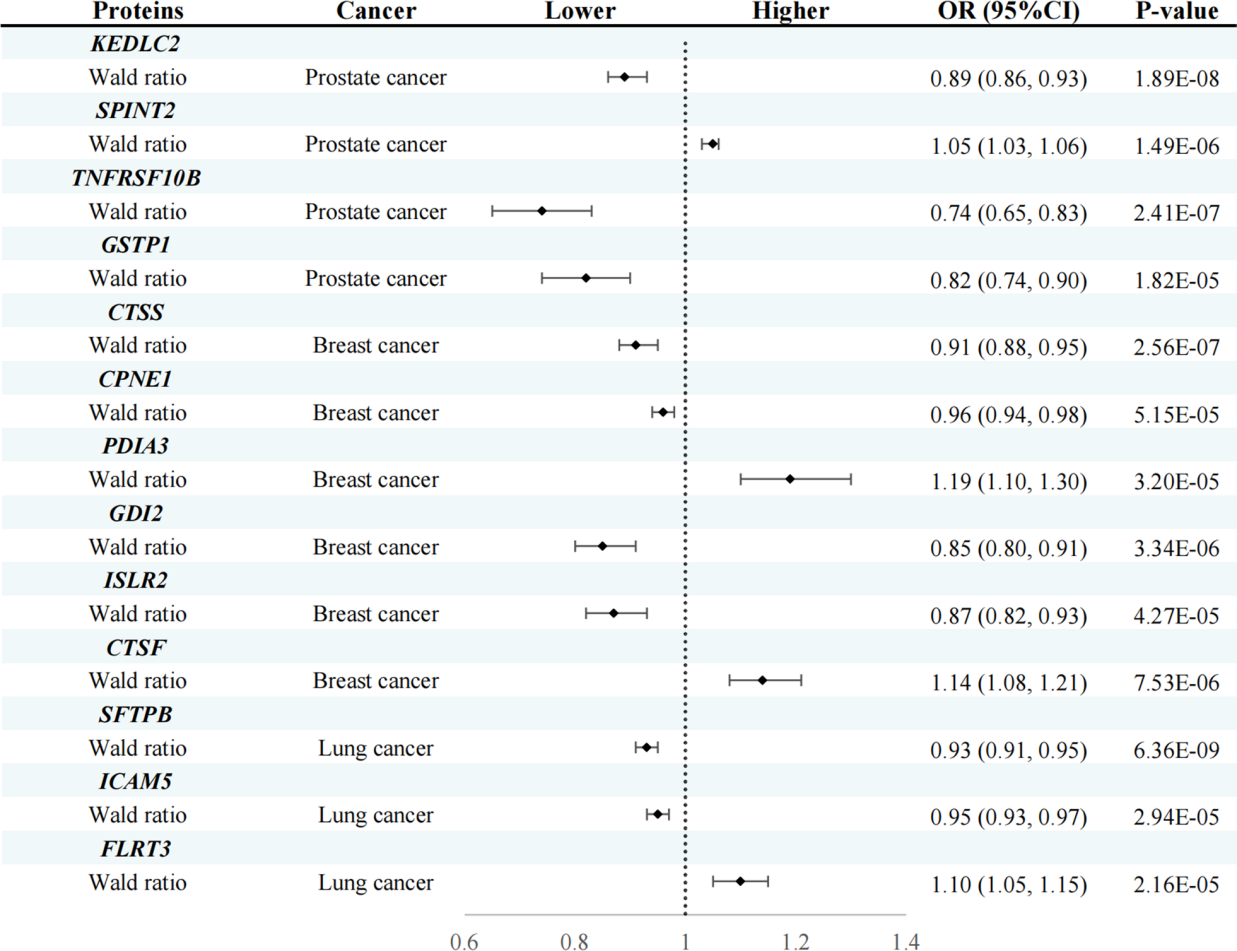
Casual effects of MR Analysis between thirteen identified proteins and three site-specific cancers

Upon excluding instances of reverse causality, our primary analysis identified thirteen plasma proteins exerting causal effects on three site-specific cancers (Fig. 2 and Table 1). Specifically, for each 1-standard deviation (SD) increase in genetically predicted protein levels, the odds ratios (ORs) for PCa were as follows: KDELC2 at 0.89 (95% CI 0.86–0.93), SPINT2 at 1.05 (95% CI 1.03–1.06), TNFRSF10B at 0.74 (95% CI 0.65–0.83), GSTP1 at 0.82 (95% CI 0.74–0.90), and CTSS at 0.91 (95% CI 0.88–0.95). For BRCa, elevated levels of CPNE1 (OR = 0.96; 95% CI 0.94–0.98), GDI2 (OR = 0.85; 95% CI 0.80–0.91), and ISLR2 (OR = 0.87; 95% CI 0.87–0.93) corresponded to a reduced risk. Conversely, increased levels of PDIA3 (OR = 1.19; 95% CI 1.10–1.30) and CTSF (OR = 1.14; 95% CI 1.08–1.21) were associated with an escalated risk of BRCa. In the case of LCa, for each 1-SD increment in genetically predicted protein levels, the ORs were 0.93 (95% CI 0.91–0.95) for SFTPB, 0.95 (95% CI 0.93–0.97) for ICAM5, and 1.10 (95% CI 1.05–1.15) for FLRT3 (Fig. 3). No heterogeneity was detected in the primary analysis (Additional file 1: Table S4).

### Colocalization analysis and phenotype scanning for cancers causal proteins

We undertook a colocalization analysis to investigate shared genetic signals between the identified proteins and three site-specific cancers: PCa, BRCa, and LCa. Notably, substantial colocalization evidence was found linking KDELC2 to PCa (Additional file 1: Fig. S1 and Table 2). Furthermore, CPNE1 demonstrated colocalization with BRCa, while SFTPB exhibited colocalization with LCa, all with substantial supporting evidence (Table 2, Additional file 1: Fig. S2, S3).

**Table 2:** Colocalization and phenotype scanning of 13 plasma proteins with cancers

Cancers	Proteins	SNP	Colocalization analysis (PPH4)	Previously reported associations
Prostate cancer	KDELC2	rs74911261	9.99E−01	Breast cancer
				Renal cell carcinoma
				Leiomyoma of uterus
				Blood cell traits
				Impedance of the body
	GSTP1	rs1695	5.71E−01	Height
				Blood cell traits
	SPINT2	rs71354995	1.26E−25	NA
	TNFRSF10B	rs4871844	6.01E−03	NA
	CTSS	rs41271951	1.12E−04	Blood protein levels
Breast cancer	CPNE1	rs12481228	8.80E−01	Impedance of the body
				Height
				Weight
				Basal metabolic rate
	ISLR2	rs2959011	4.20E−01	Hypertension
	PDIA3	rs3110081	1.07E−02	NA
	GDI2	rs2890364	1.83E−01	NA
	CTSF	rs1791679	4.93E−05	NA
Lung cancer	SFTPB	rs1130866	9.51E−01	Granulysin
	ICAM5	rs281439	3.68E−02	Lymphocyte count
				Intercellular adhesion molecule 1
	FLRT3	rs11908097	3.18E−01	NA

SNP: single nucleotide polymorphism; PPH4: posterior probability of hypothesis 4

In the phenotype scanning phase, based on the genome-wide significance threshold (P < 5 × 10^−8^), we observed KDELC2 to be associated with various cancers, such as breast cancer, renal cell carcinoma, and uterine leiomyoma, which suggested that KDELC2 may be deficient in specificity for PCa diagnosis. In addition, KDELC2 demonstrated associations with blood cell traits and body impedance. GSTP1 showed associations with height and blood cell traits, while CPNE1 was linked to body impedance, height, weight, and basal metabolic rate. ISLR2 was found to be associated with hypertension, and SFTPB displayed an association with Granulysin (Table 2). However, we uncovered no direct evidence linking these phenotypes to the specific influence on PCa, BRCa, or LCa (Additional file 1: Table S5).

### External validation of causal proteins for cancers

In the external validation phase, we corroborated the MR results using both the same-variant and significant-variant approaches from additional datasets. By deploying the same-variant and significant-variant plasma proteins acquired from Ferkingstad et al. [17] as genetic instruments, we effectively replicated the causal relationships between KDELC2, SPINT2, CTSS, and TNFRSF10B with PCa in the UK Biobank. Likewise, we successfully replicated the causal relationships between CPNE1, PDIA3, and CTSF with BRCa in the UK Biobank. Regrettably, the proteins identified for LCa failed to replicate successfully during the external validation phase. However, it's noteworthy that the associations for the remainder of the identified proteins displayed consistent directional trends in the replication analysis (Additional file 1: Table S6).

### Causal protein's druggability and its association with current medications

In an effort to deduce the potential mechanisms of action for the identified drug targets, we queried DrugBank for the targets of selected cancer-related drugs (Additional file 1: Table S7) and devised a PPI network between the identified proteins and the targets of cancer-related drugs using STRING (Additional file 1: Fig. S4A–C). The resulting PPI network exposed interactions between two causative proteins (TNFRSF10B, GSTP1) and the targets of four drugs currently used in PCa treatment, of which TNFRESF10B-CASP8, GSTP1-CYP17A1, and GSTP1-AR were considered strong interactions (Additional file 1: Fig. S4A). Specifically, TNFRSF10B demonstrated a robust interaction with its target, Caspase-8 (CASP8), also a target for the drug Bardoxolone. GSTP1 revealed strong interactions with its target, the Androgen receptor (AR), a common target for Apalutamide and Enzalutamide (Additional file 1: Fig. S4A). For BRCa, the PPI network unveiled a robust interaction between PDIA3 and the target of the drug Neratinib, Epidermal growth factor receptor (EGFR) (Additional file 1: Fig. S4B).

In addition, we examined drug databases for potential drugs targeting the proteins identified for PCa, BRCa and LCa treatments, such as Bioymifi (a binder of TNFRSF10B) for PCa, alpha-Tocopherol succinate (an inhibitor of GSTP1) for PCa, and Theophylline for BRCa. A summary of both investigational and approved medications targeting the identified proteins is available in Additional file 1: Table S8. Despite the known associations, no medications targeting the proteins identified for LCa have been documented in DrugBank.

Finally, guided by our colocalization analysis, external validation, and PPI network, we categorized the proteins into four distinct target groups (Additional file 1: Table S9).

## Discussion

To our knowledge, this study is the first to scrutinize the causal associations between 732 plasma proteins and 8 site-specific cancers by employing MR and Bayesian colocalization analyses. We were able to identify thirteen plasma proteins linked with three site-specific cancers. MR analysis uncovered five proteins (KDELC2, SPINT2, TNFRSF10B, GSTP1, and CTSS) associated with PCa, five proteins (CPNE1, PDIA3, GDI2, ISLR2, and CTSF) linked with BRCa, and three proteins (SFTPB, ICAM5, and FLRT3) related to LCa. Unfortunately, applying Bonferroni correction resulted in scant evidence of associations between plasma proteins and the remaining five site-specific cancers. During the external validation stage, four out of the five proteins associated with PCa (KDELC2, SPINT2, TNFRSF10B, and CTSS) and three out of the five proteins linked with BCa (CPNE1, PDIA3, and GDI2) were successfully replicated using similar approaches in the UK Biobank, further bolstering the reliability of the potential drug targets identified in this study.

In this research, we employed a multitude of methods to search for novel drug targets within plasma proteins for site-specific cancers. To mitigate the effects of reverse causality and horizontal pleiotropy on causal relationships, we utilized Steiger filtering to ensure the directionality of causal effects, while bidirectional MR analysis was further leveraged to scrutinize potential reverse causality. To minimize the impact of horizontal pleiotropy, we restricted our use of plasma protein cis-pQTLs as instruments. Bayesian colocalization analysis was also incorporated to further eliminate biases, and we classified the identified proteins into four distinct target groups based on their PPH4 values (Additional file 1: Table S9). Phenotype scanning revealed that seven out of the thirteen identified proteins (KDELC2, GSTP1, CTSS, CPNE1, ISLR2, SFTPB, and ICAM5) were associated with other traits, but none of these traits were likely to bias the associations between identified proteins and cancers.

In addition, we created a PPI network to explore the associations between identified proteins and known drug targets, with the aim to screen and prioritize potential drug targets. For PCa, we identified KDELC2 as a tier 1 target, TNFRSF10B as a tier 2 target, and SPINT2, CTSS, and GSTP1 as tier 3 targets. For BRCa, CPNE1 was identified as a tier 1 target, PDIA3 as a tier 2 target, and GDI2 as tier 3 targets. For LCa, SFTPB was identified as a tier 3 target (Additional file 1: Table S9).

KDELC2, also known as Protein O-glucosyltransferase 3 or Poglut 3, is part of the KDEL-containing protein family, which is known for its critical roles in the control of protein quality and trafficking within the endoplasmic reticulum [37, 38]. Notably, these proteins govern a range of signaling pathways and biological processes through their involvement in protein O-glucosylation modifications [39]. The Notch signaling pathway has been identified as significant in PCa [40, 41]. Zhang et al. [42] suggested that Notch signaling inhibits the progression of cancer by upregulating the expression of genes of the Phosphatase and tensin homolog (PTEN). Studies [43] have shown that deletion of the gene for PTEN and dysregulation of PI3K/m TOR signaling lead to the transformation of prostate normal cells to malignant cells in vitro and in a mouse model, while the Notch pathway is able to inhibit the transformation of prostate cells to malignant cells by up-regulating the expression of the gene for PTEN or by affecting PI3K/m TOR signaling. In addition, J Shou et al. [44] discovered that the proliferative capacity of prostate cancer cells was inhibited by sustained activation of the Notch1 functional fragment ICN in the prostate cancer cell lines PC3, DU145, and LNCaP, which further illustrates the regulatory role of the Notch receptor in prostate cancer cells. Several studies revealed [39, 45] that KDELC2 plays an important role in the activation of the notch signaling pathway. Specifically, Notch receptors consist of the Notch intracellular structural domain (NICD) and the Notch extracellular structural domain (NECD). While the main part of NECD mainly consists of 36 epidermal growth factor (EGF)-like motifs [46, 47], NECD contains enriched surface-modified O-linked glycans, such as O-glucose (O-Glc), O-fucose (O-Fuc), and O-GlcNAc [47, 48]. KDECL2 facilitates the transfer of O-glucose to Notch 1 EGF11 and Notch 3 EGF10 [39, 45], and such transfers enhance the Notch receptor-ligand connection between the Notch receptor and ligand, activating Notch signaling [39, 45]. Combining the evidence above, we theorize that KDELC2 may regulate the Notch signaling pathway through O-glucosylation modifications of Notch receptors, thus influencing the proliferation of prostate cancer cells [38, 45]. Moreover, KDELC2 might also contribute to the regulation of specific prostate cancer suppressor cells or molecules, playing a pivotal role in the apoptosis of cancer cells [49]. Presently, there is limited research and information available on the explicit role and targets of KDELC2 in the regulation of PCa. Nevertheless, within our study, KDELC2 is the sole tier 1 target identified for PCa, which implies that it could serve as a novel drug target for PCa. Still, more research is required to uncover its specific role in prostate cancer.

Tumor necrosis factor receptor superfamily member 10B (TNFRSF10B), as a member of the tumor necrosis factor receptor superfamily, also referred to as TNF-related apoptosis-inducing ligand 2 (TRAIL-R2) or death receptor 5 (DR5), has been identified as a protective tier 2 target for PCa in this study. Consistent with our findings, several studies have demonstrated that therapies aimed at TNFRSF10B show promising anti-tumor activity in PCa and have low cytotoxicity to normal cells [50–52]. Specifically, TRAIL, when bound to TNFRSF10B, instigates programmed cell death, aids in the recruitment of adapter proteins, promotes the assembly of the death-inducing signaling complex (DISC), and subsequently triggers the activation of the caspase cascade [53]. Although TNFRSF10B didn't meet the PPH4 significance threshold in the colocalization analysis, the PPI network shows TNFRSF10B exhibiting strong interactions with the therapeutic target (caspase-8) of Bardoxolone. Bardoxolone, as a novel Nrf-2 inducer, has been shown to improve the efficacy of enzalutamide in resistant prostate cancer [54]. Therefore, TNFRSF10B also shows promise as a target for prostate cancer.

Copine-1, a calcium-dependent phospholipid-binding protein encoded by the CPNE1 gene, is part of the Copine family of proteins. They’re known for their C2 domains and involvement in diverse cellular processes such as signal transduction and membrane trafficking [55]. The connection between CPNE1 and BRCa risk isn't entirely clear, as prior studies have reported inconsistent results [56]. This inconsistency could be due to differences in BRCa molecular subtyping used in our analysis compared to traditional epidemiological studies, or it could underscore the limitations in conventional epidemiological studies in adjusting for confounding factors and reverse causality. In our study, CPNE1 was classified as a tier 1 target, implying a high potential for CPNE1 to be a drug target for BRCa Nonetheless, more experimentation is necessary to establish the association directionality between CPNE1 and BRCa. We also discovered suggestive evidence of an association between Protein disulfide isomerase A3 (PDIA3) and BRCa. PDIA3, a disulfide oxidoreductase, and isomerase located in the endoplasmic reticulum, is supported by previous studies aligning with our findings [57, 58]. Notably, the suppression of PDIA3 transcripts in human breast cancer cell lines was found to inhibit cell proliferation and increase cell sensitivity toward chemotherapy or radiation treatment [59]. PDIA3 was also essential for the propensity of a metastatic subline of human MDA-MB-231 breast cancer cells for bone metastasis in a nude mouse model [60]. In the PPI network, PDIA3 interacts with the target of Neratinib, the Epidermal growth factor receptor (EGFR) (Additional file 1: Fig. S4B). This interaction suggests that PDIA3 might promote the growth and proliferation of breast cancer cells by impacting the tyrosine kinase activity of human Epidermal Growth Factor Receptor 2 (HER2). Therefore, we hypothesize that combining PDIA3 inhibitors with tyrosine kinase inhibitors (such as Neratinib, Trastuzumab, and Pertuzumab) could enhance the inhibitory effect of tyrosine kinase inhibitors on breast cancer cells.

The remaining proteins identified in our study were categorized as tier 3 or 4 targets, indicating their potential utility as cancer drug targets. However, additional experimental validation is necessary to confirm the reliability of these potential drug targets in tiers 3 and 4. These include tiers 3 targets for PCa such as SPINT2, GSTP1, and CTSS, tier 3 target (GDI2) for BRCa, and tiers 4 targets (ISLR2 and CTSF) for BRCa, tier 3 target (SFTPB) and tier 4 targers (ICAM5, and FLRT3) for LCa.

Interestingly, we noticed that in the PPI network, GSTP1 interacts with the targets of three currently used drugs for PCa. GSTP1 is a specific subtype of glutathione S-transferases (GSTs), also known as "GST pi 1". The activity and expression levels of GSTP1 may be influenced by genetic variations and environmental factors, impacting cellular detoxification capacity and antioxidant defense mechanisms [61]. In the context of cancer, GSTP1 has been associated with susceptibility to certain tumors and responsiveness to chemotherapy drugs [62]. Prior proteomic studies corroborate our finding [63, 64] that downregulation of GSTP1 is associated with an increased risk of PCa, highlighting its potential as a PCa inhibitor [62] and a promising drug target. Moreover, in the PPI network, GSTP1 interacts with the targets of several drugs for PCa, namely, abiraterone, enzalutamide, apalutamide, and uracil (with target proteins CYP17A1, androgen receptor, DPYD). This interaction suggests that drugs designed to target GSTP1 might inhibit androgen synthesis or boost the efficacy of other drugs, such as 5-fluorouracil, in inhibiting DNA synthesis for the treatment of prostate cancer.

Even though our study presents some insightful findings, it's crucial to acknowledge its several limitations. Chiefly among these is the issue of data limitation. Because of this, our focus was on the overall data for each cancer type without considering the various subtypes of cancer. This limitation underscores the need for future research to thoroughly dissect the roles of these proteins in specific subtypes of cancer. Additionally, we sourced our protein data from various studies for our analysis. Despite the fact that these variations in measurements across different studies could introduce bias, we tried to mitigate this by using all circulating protein data based on aptamer technology. Still, another limitation we need to consider is the specificity of our research population. Since our research was primarily on European populations, the results might not be generalizable to other racial or ethnic groups. Moreover, our study was constrained by the limited number of genetic instruments available, making it impossible to conduct sensitivity analyses using additional Mendelian Randomization methods for most identified targets. However, using cis-pQTLs as instruments could potentially decrease the risk of horizontal pleiotropy. Furthermore, all SNPs included in the study had F-statistic values greater than 10, indicating that weak instrument bias is highly unlikely. However, it is essential to note that while our study provides preliminary evidence of potential associations between drug targets and cancer, these findings should be further validated through comprehensive research. Regrettably, due to funding limitations, we were not able to conduct in-depth biological experiments to unearth the specific mechanisms through which these drug targets might influence tumors. As a solution, future research could potentially involve animal models and cell line experiments to provide more substantial validation of our findings. Lastly, our study was primarily focused on proteins with available index pQTL signals at the genome-wide significance threshold, which could potentially overlook some drug targets.

## Conclusions

MR and Bayesian colocalization analyses were combined in this study to identify thirteen potential drug targets specific to three site-specific cancers. Additionally, external validation and PPI network analysis classified these proteins into four distinct target groups. The top-tier targets (Tier 1 and 2) are the most promising candidates for therapeutic drug development. Examples include KDELC2 and TNFRSF10B for PCa and CPNE1 and PDIA3 for BRCa. However, these findings require further validation through future biological experiments.

### Supplementary Information

**Additional file 1: Table S1.** Instrumental variables of plasma proteins used in MR analysis. **Table S2.** The sources for all statistical summary datasets used in this study. **Table S3.** Casual effects between 3 site-specific cancers and 13 identified proteins in the bidirectional MR analysis. **Table S4.** The results of heterogeneity analysis. **Table S5.** Investigating the Previous Genome-Wide Significant Associations of SNPs as Genetic Instruments for Potential Causal Proteins. **Table S6.** Genetic Instruments for Drug Targets Validated in External Validation. **Table S7.** Current anticancer medications and corresponding targets for cancer treatment. **Table S8.** Current medications targeting potential causal proteins. **Table S9.** Distinct target groups of identified proteins. **Figure S1.** Colocalization Analysis of Plasma Proteins for prostate cancer. The diamond purple points represent SNPs that exhibit the lowest combined P-value in both protein GWAS and cancer GWAS analyses. **Figure S2.** Colocalization Analysis of Plasma Proteins for breast cancer.The diamond purple points represent SNPs that exhibit the lowest combined P-value in both protein GWAS and cancer GWAS analyses. **Figure S3.** Colocalization Analysis of Plasma Proteins for lung cancer. The diamond purple points represent SNPs that exhibit the lowest combined P-value in both protein GWAS and cancer GWAS analyses. **Figure S4.** Protein–protein interaction network between identified proteins and cancer-associated medication targets. Green circles represent the targets of current medications for prostate cancer; Red circles represent potential drug targets identified in this study; Blue circles represent the current medications targets that interact with potential drug targets. R-detailed code.

## Data Availability

GWAS summary statistics utilized in this research can be accessed from the IEU OpenGWAS database (https://gwas.mrcieu.ac.uk/) or from the primary research website. Further details are available in Additional file 1: Table S2.

## Abbreviations

cis-MR: Cis-Mendelian randomization
pQTL: Protein quantitative trait loci
GWAS: Genome-wide association studies
PCa: Prostate cancer
BRCa: Breast cancer
LCa: Lung cancer
CCa: Colorectal cancer
BLCa: Bladder cancer
OCa: Ovarian cancer
KCa: Kidney cancer
GCa: Gastric cancer
MHC: Major histocompatibility complex
LD: Linkage disequilibrium
SNP: Single nucleotide polymorphism
IVW: Inverse Variance Weighted
PPH4: Posterior probability of hypothesis 4
PPI: Protein–protein interaction
SD: Standard deviation
ORs: Odds ratios
AR: Androgen receptor
CASP8: Caspase-8
EGFR: Epidermal growth factor receptor
TRAIL-R2: TNF-related apoptosis-inducing ligand 2
DR5: Death receptor 5
DISC: Death-inducing signaling complex
HER2: Human epidermal growth factor receptor 2
GSTs: Glutathione S-transferases
KDELC2: KDEL (Lys-Asp-Glu-Leu) containing 2, isoform CRA_a
PTEN: Phosphatase and tensin homolog
SPINT2: Kunitz-type protease inhibitor 2
TNFRSF10B: Tumor necrosis factor receptor superfamily member 10B
GSTP1: Glutathione S-transferase P
CTSS: Cathepsin S
CPNE1: Copine-1
PDIA3: Protein disulfide-isomerase A3
GDI2: Rab GDP dissociation inhibitor
ISLR2: Immunoglobulin superfamily containing leucine-rich repeat protein 2
CTSF: Cathepsin F
SFTPB: Pulmonary surfactant-associated protein B
ICAM5: Intercellular adhesion molecule 5
FLRT3: Leucine-rich repeat transmembrane protein FLRT3
