# Supplementary figures and images for: Proteome-wide mendelian randomization study implicates therapeutic targets in common cancers
Source: J Transl Med. 2023 Sep 21;21:646. doi: 10.1186/s12967-023-04525-5 (PMC10512580; doi:10.1186/s12967-023-04525-5)

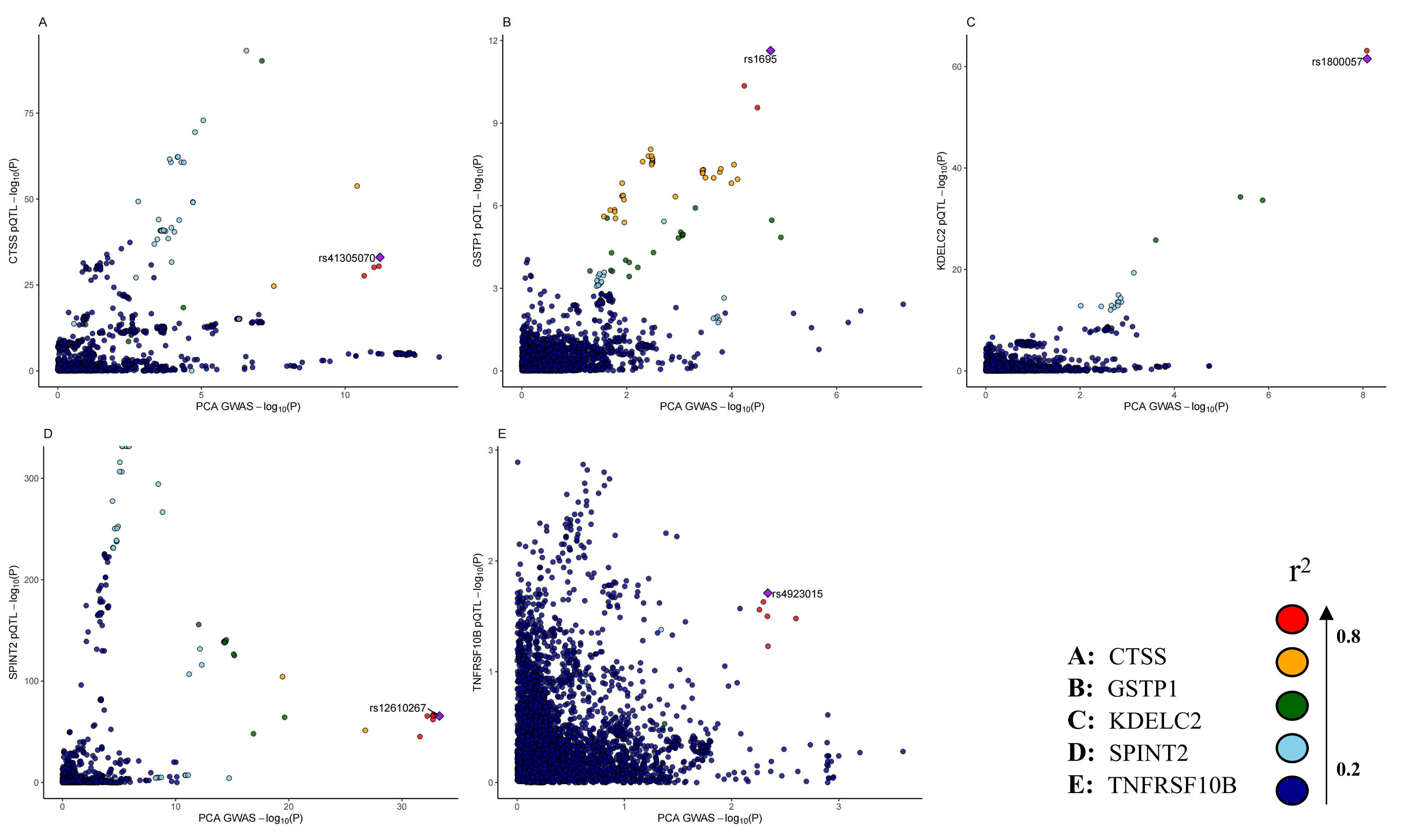

Supplement: Supplementary file 1 — Additional file 1: Table S1. Instrumental variables of plasma proteins used in MR analysis. Table S2. The sources for all statistical summary datasets used in this study. Table S3. Casual effects between 3 site-specific cancers and 13 identified proteins in the bidirectional MR analysis. Table S4. The results of heterogeneity analysis. Table S5. Investigating the Previous Genome-Wide Significant Associations of SNPs as Genetic Instruments for Potential Causal Proteins. Table S6. Genetic Instruments for Drug Targets Validated in External Validation. Table S7. Current anticancer medications and corresponding targets for cancer treatment. Table S8. Current medications targeting potential causal proteins. Table S9. Distinct target groups of identified proteins. Figure S1. Colocalization Analysis of Plasma Proteins for prostate cancer. The diamond purple points represent SNPs that exhibit the lowest combined P-value in both protein GWAS and cancer GWAS analyses. Figure S2. Colocalization Analysis of Plasma Proteins for breast cancer.The diamond purple points represent SNPs that exhibit the lowest combined P-value in both protein GWAS and cancer GWAS analyses. Figure S3. Colocalization Analysis of Plasma Proteins for lung cancer. The diamond purple points represent SNPs that exhibit the lowest combined P-value in both protein GWAS and cancer GWAS analyses. Figure S4. Protein–protein interaction network between identified proteins and cancer-associated medication targets. Green circles represent the targets of current medications for prostate cancer; Red circles represent potential drug targets identified in this study; Blue circles represent the current medications targets that interact with potential drug targets. R-detailed code. [file 12967_2023_4525_MOESM1_ESM.zip › Figure S1.tif]

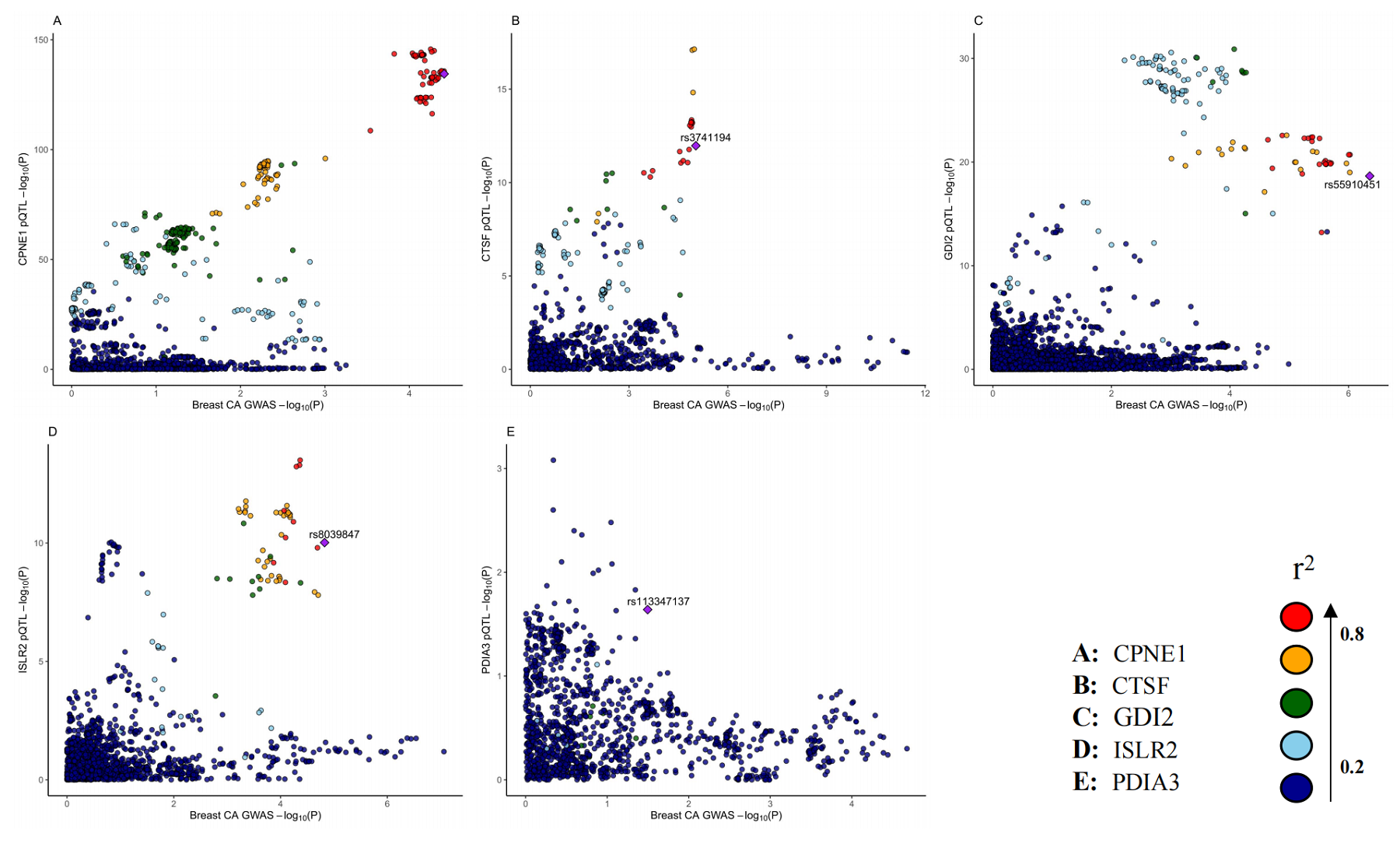

Supplement: Supplementary file 1 — Additional file 1: Table S1. Instrumental variables of plasma proteins used in MR analysis. Table S2. The sources for all statistical summary datasets used in this study. Table S3. Casual effects between 3 site-specific cancers and 13 identified proteins in the bidirectional MR analysis. Table S4. The results of heterogeneity analysis. Table S5. Investigating the Previous Genome-Wide Significant Associations of SNPs as Genetic Instruments for Potential Causal Proteins. Table S6. Genetic Instruments for Drug Targets Validated in External Validation. Table S7. Current anticancer medications and corresponding targets for cancer treatment. Table S8. Current medications targeting potential causal proteins. Table S9. Distinct target groups of identified proteins. Figure S1. Colocalization Analysis of Plasma Proteins for prostate cancer. The diamond purple points represent SNPs that exhibit the lowest combined P-value in both protein GWAS and cancer GWAS analyses. Figure S2. Colocalization Analysis of Plasma Proteins for breast cancer.The diamond purple points represent SNPs that exhibit the lowest combined P-value in both protein GWAS and cancer GWAS analyses. Figure S3. Colocalization Analysis of Plasma Proteins for lung cancer. The diamond purple points represent SNPs that exhibit the lowest combined P-value in both protein GWAS and cancer GWAS analyses. Figure S4. Protein–protein interaction network between identified proteins and cancer-associated medication targets. Green circles represent the targets of current medications for prostate cancer; Red circles represent potential drug targets identified in this study; Blue circles represent the current medications targets that interact with potential drug targets. R-detailed code. [file 12967_2023_4525_MOESM1_ESM.zip › Figure S2.tif]

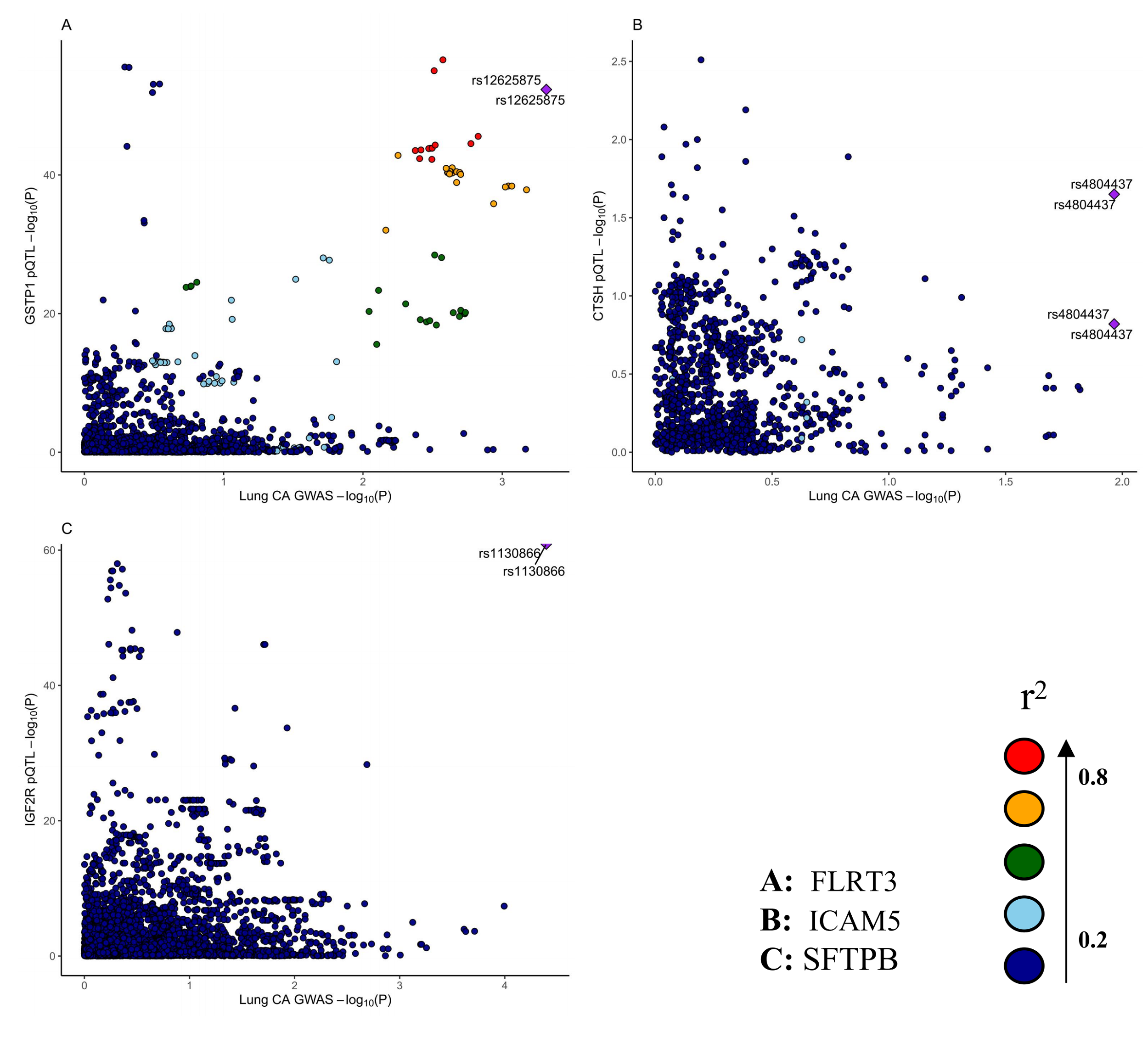

Supplement: Supplementary file 1 — Additional file 1: Table S1. Instrumental variables of plasma proteins used in MR analysis. Table S2. The sources for all statistical summary datasets used in this study. Table S3. Casual effects between 3 site-specific cancers and 13 identified proteins in the bidirectional MR analysis. Table S4. The results of heterogeneity analysis. Table S5. Investigating the Previous Genome-Wide Significant Associations of SNPs as Genetic Instruments for Potential Causal Proteins. Table S6. Genetic Instruments for Drug Targets Validated in External Validation. Table S7. Current anticancer medications and corresponding targets for cancer treatment. Table S8. Current medications targeting potential causal proteins. Table S9. Distinct target groups of identified proteins. Figure S1. Colocalization Analysis of Plasma Proteins for prostate cancer. The diamond purple points represent SNPs that exhibit the lowest combined P-value in both protein GWAS and cancer GWAS analyses. Figure S2. Colocalization Analysis of Plasma Proteins for breast cancer.The diamond purple points represent SNPs that exhibit the lowest combined P-value in both protein GWAS and cancer GWAS analyses. Figure S3. Colocalization Analysis of Plasma Proteins for lung cancer. The diamond purple points represent SNPs that exhibit the lowest combined P-value in both protein GWAS and cancer GWAS analyses. Figure S4. Protein–protein interaction network between identified proteins and cancer-associated medication targets. Green circles represent the targets of current medications for prostate cancer; Red circles represent potential drug targets identified in this study; Blue circles represent the current medications targets that interact with potential drug targets. R-detailed code. [file 12967_2023_4525_MOESM1_ESM.zip › Figure S3.tif]

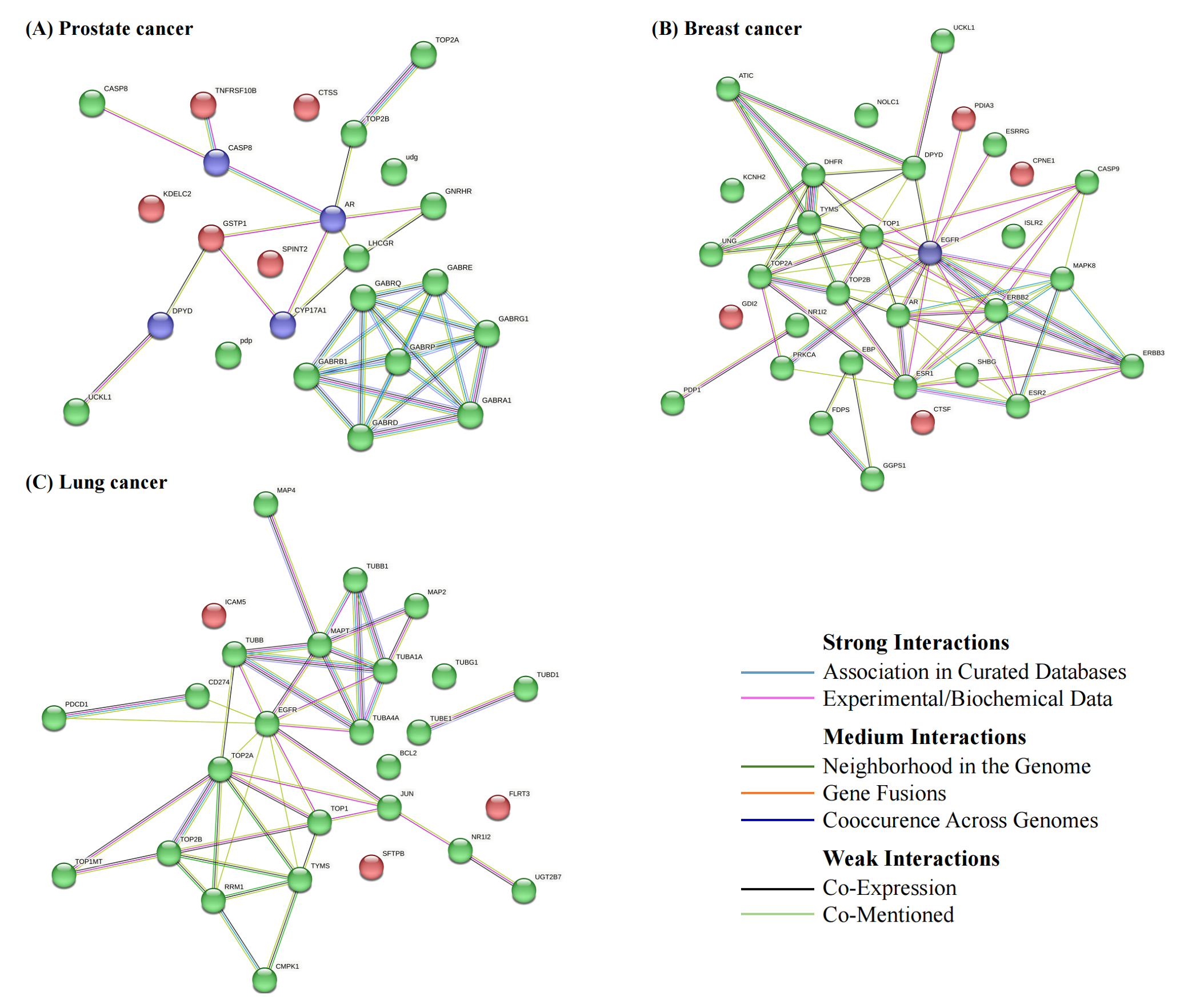

Supplement: Supplementary file 1 — Additional file 1: Table S1. Instrumental variables of plasma proteins used in MR analysis. Table S2. The sources for all statistical summary datasets used in this study. Table S3. Casual effects between 3 site-specific cancers and 13 identified proteins in the bidirectional MR analysis. Table S4. The results of heterogeneity analysis. Table S5. Investigating the Previous Genome-Wide Significant Associations of SNPs as Genetic Instruments for Potential Causal Proteins. Table S6. Genetic Instruments for Drug Targets Validated in External Validation. Table S7. Current anticancer medications and corresponding targets for cancer treatment. Table S8. Current medications targeting potential causal proteins. Table S9. Distinct target groups of identified proteins. Figure S1. Colocalization Analysis of Plasma Proteins for prostate cancer. The diamond purple points represent SNPs that exhibit the lowest combined P-value in both protein GWAS and cancer GWAS analyses. Figure S2. Colocalization Analysis of Plasma Proteins for breast cancer.The diamond purple points represent SNPs that exhibit the lowest combined P-value in both protein GWAS and cancer GWAS analyses. Figure S3. Colocalization Analysis of Plasma Proteins for lung cancer. The diamond purple points represent SNPs that exhibit the lowest combined P-value in both protein GWAS and cancer GWAS analyses. Figure S4. Protein–protein interaction network between identified proteins and cancer-associated medication targets. Green circles represent the targets of current medications for prostate cancer; Red circles represent potential drug targets identified in this study; Blue circles represent the current medications targets that interact with potential drug targets. R-detailed code. [file 12967_2023_4525_MOESM1_ESM.zip › Figure S4.tif]
